# Tomato disease object detection method combining prior knowledge attention mechanism and multiscale features

**DOI:** 10.3389/fpls.2023.1255119

**Published:** 2023-10-09

**Authors:** Jun Liu, Xuewei Wang

**Affiliations:** Shandong Provincial University Laboratory for Protected Horticulture, Weifang University of Science and Technology, Weifang, China

**Keywords:** complex background, tomato diseases, prior knowledge, attention mechanism, multi-scale features, object detection

## Abstract

To address the challenges of insufficient accuracy in detecting tomato disease object detection caused by dense target distributions, large-scale variations, and poor feature information of small objects in complex backgrounds, this study proposes the tomato disease object detection method that integrates prior knowledge attention mechanism and multi-scale features (PKAMMF). Firstly, the visual features of tomato disease images are fused with prior knowledge through the prior knowledge attention mechanism to obtain enhanced visual features corresponding to tomato diseases. Secondly, a new feature fusion layer is constructed in the Neck section to reduce feature loss. Furthermore, a specialized prediction layer specifically designed to improve the model’s ability to detect small targets is incorporated. Finally, a new loss function known as A-SIOU (Adaptive Structured IoU) is employed to optimize the performance of the model in terms of bounding box regression. The experimental results on the self-built tomato disease dataset demonstrate the effectiveness of the proposed approach, and it achieves a mean average precision (mAP) of 91.96%, which is a 3.86% improvement compared to baseline methods. The results show significant improvements in the detection performance of multi-scale tomato disease objects.

## 1 Introduction

Due to the ongoing expansion of tomato cultivation areas and limited arable land, a growing contradiction has emerged between the two. As a result, consecutive cropping of tomatoes has become prevalent, resulting in an increase in the variety and complexity of tomato diseases. According to relevant studies, there are currently more than thirty types of fungal diseases alone affecting tomatoes worldwide (Widjaja et al., 2022). In China, there are several prevalent and influential tomato diseases that significantly impact tomato cultivation. These include early blight, late blight, bacterial spot, gray leaf spot, gray mold, leaf mold, yellow leaf curl virus, mosaic virus, canker, and anthracnose (Liu and Wang, 2020).

Tomato diseases have become a prominent issue in China, leading to a reduction in yield of approximately 10%. In areas severely impacted by these diseases, complete crop failure has been observed (Thangaraj et al., 2022). Tomato diseases not only result in a reduction in tomato yield but also pose risks to storage and transportation due to the contamination of infected fruits. As a result, the efficient diagnosis and control of tomato diseases have emerged as critical concerns in tomato production.

During the early stages of tomato diseases, farmers often neglect to assess and manage these diseases due to their unclear symptoms. This oversight frequently results in missing the optimal period for disease prevention and control. As the tomato diseases progress and become severe, the application of a large amount of fungicides proves to be ineffective. Another group of farmers faces challenges in assessing whether their tomatoes are infected and lacks the ability to distinguish the severity of the diseases. Consequently, they resort to extensively using fungicides for disease prevention and control. Unfortunately, prolonged implementation of such practices leads to the excessive use of fungicides, posing risks to environmental safety and human health (Moussafir et al., 2022). Therefore, there is an increasing demand for timely and effective identification, detection, and precise application of treatments for tomato diseases, making it a prominent research topic in recent years.

Through the long-term collaborative efforts of agricultural and plant protection scholars, notable advancements have been made in the domain of tomato disease control and prevention in China. Commonly employed methods include empirical analysis based on observable symptoms and physicochemical analysis. However, when it comes to large-scale detection, the limited number of experts hinders their ability to provide real-time monitoring of tomato diseases across the entire production line. Additionally, expert judgments may be swayed by various influential elements, including weather conditions and theoretical knowledge, making it challenging to timely and accurately assess the occurrence of tomato diseases in actual production. Moreover, the physicochemical analysis of tomato diseases requires a significant number of specialized technicians, is time-consuming, and poses the risk of secondary transmission of diseases due to human activities. Consequently, there is an urgent need to explore and develop rapid, accurate, non-destructive, and environmentally-friendly methods for detecting tomato diseases, which has become a key research focus.

The development of modern computer technology has led to increasingly refined applications of new artificial intelligence information in agriculture. Over the course of more than 30 years of progress in artificial intelligence, intelligent diagnosis has been implemented in various aspects of crop cultivation management, plant protection, crop breeding, and agricultural planting decisions (Misra et al., 2020). These advancements have greatly enhanced the efficiency and accuracy of agricultural practices. Additionally, the integration of artificial intelligence and image recognition enables rapid, accurate, and non-destructive identification and diagnosis of diseases. Image detection primarily relies on cameras and other devices to capture information on crop diseases, thereby reducing the need for human observation (Mohammad-Razdari et al., 2022). By leveraging digital image processing, healthy and diseased crops can be identified and classified accurately.

However, there is still significant room for improvement in the actual tomato disease detection process, as the current detection accuracy and algorithm processing speed do not meet the requirements of real-world farming scenarios (David et al., 2021) (Karthik et al., 2020). Several challenges contribute to this limitation. Firstly, there is an imbalance in the number of samples available for different tomato diseases (Abbas et al., 2021). This scarcity of samples makes it difficult to obtain an adequate representation of various diseases, which in turn hampers model training and severely restricts the learning capacity of deep learning models. Secondly, tomato disease detection possesses unique characteristics. The natural background of tomato diseases is complex and diverse, and different types of diseases exhibit distinct characteristics (Gonzalez-Huitron et al., 2021). Even with a sufficient number of tomato disease samples, relying solely on visual features makes accurate identification challenging (Huang et al., 2023). In contrast, humans possess the ability to quickly learn and assimilate new knowledge based on their accumulated experiences, which is referred to as prior knowledge. This suggests that incorporating prior knowledge of tomato diseases into tomato disease detection is essential to enhance learning efficiency (Diligenti et al., 2017). Therefore, it is crucial to integrate deep learning models with prior knowledge in the field of tomato disease detection in order to overcome these challenges.

Applying existing deep learning models directly to tomato disease detection tasks makes it challenging to accurately differentiate the distinctive features of different diseases. This limitation often leads to a significant number of misclassifications or omissions. Consequently, the integration of deep learning models with prior knowledge and the improvement of tomato disease detection accuracy through a collaborative “data model knowledge” approach have become common challenges faced by both the agricultural and academic communities. To address the lack of explicit expression of objective prior knowledge in deep learning models and the imbalanced distribution of disease samples, this research aims to combine deep learning models with disease prior knowledge. The study focuses on tomato diseases occurring in complex backgrounds, considering the complexity of tomato disease data and utilizing prior knowledge. As a result, a tomato disease object detection method that integrates a prior knowledge attention mechanism and multi-scale features is proposed.

## 2 Related work

### 2.1 Object detection

Object detection technology encompasses multi-object classification and localization as its primary tasks. It is not only responsible for determining whether the detection area contains target objects but also for marking these targets with bounding boxes. Over the past few years, the remarkable and swift progress in the field of computer technology has been noteworthy, coupled with advancements in convolutional neural networks, has significantly propelled object detection technology forward. As a result, it has found extensive applications in diverse fields including traffic monitoring and tracking, video surveillance and security alert systems, drone scene analysis, and robotic vision (Zou et al., 2023).

Object detection technology can be categorized into traditional approaches and those based on deep learning. Traditional approaches are founded upon the dependence of manual feature extraction and conventional classifiers for object classification. However, they are plagued by problems like weak feature representation, suboptimal accuracy and inadequate real-time performance. In contrast, the rapid growth of big data and computing hardware has led to the widespread adoption and acceptance of deep learning in the field of object detection. It excels in feature extraction, which brings notable benefits such as enhanced detection accuracy and accelerated processing speed. As a result, it is gradually emerging as the dominant technology in the field of computer vision (Zhao et al., 2019). This technology has shown great potential in various applications, including tomato disease detection, where accurate and efficient identification of diseases is crucial for effective disease management in agriculture.

### 2.2 Plant disease object detection method in laboratory environment

Zhang et al. (2020) developed an enhanced iteration of the Faster-RCNN algorithm, specifically tailored for the identification of healthy tomato leaves and the detection of four different diseases. Instead of using VGG16, they utilized ResNet101 as the feature extractor. The experimental findings substantiated that the enhanced detection approach yielded a 2.71% increase in accuracy, while also providing faster detection speed. Wang et al. (2021) conducted experiments using the PlantVillage dataset and found that the DBA_SSD algorithm outperformed other object detection algorithms. However, It is important to highlight that the images employed in these studies was primarily captured in controlled laboratory environments. In such environments, the samples benefitted from sufficient lighting, simple and uniform backgrounds, and carefully controlled shooting angles. Moreover, agricultural experts screened and annotated the samples, resulting in more distinct disease features. In contrast, images collected in natural environments are significantly more complex. Various uncontrollable factors such as environmental location, weather conditions, and shooting angles pose challenges, including uneven lighting, shadow occlusion, overlapping leaves, and complex backgrounds (Liu and Wang, 2021). Consequently, object detection models trained solely under laboratory conditions are inadequate for real-world natural environments and fail to fully meet the production needs of farmers. The performance of these models can be affected by various factors such as lighting conditions, variations in plant appearance, and diverse backgrounds in the field. Therefore, it is crucial to train object detection models using datasets that encompass a wide range of real-world scenarios, including different weather conditions, growth stages, and farming practices.

### 2.3 Object detection method for plant diseases in real natural environments

In real natural environments, the complexity of the image sample backgrounds adds to the difficulty of the detection task. Training an effective deep learning model for disease object detection necessitates a substantial amount of data. Consequently, this task has garnered considerable attention and become a significant challenge in current research endeavors.

Fuentes et al. (2017) conducted fine-tuning of classical models using transfer learning on a self-built dataset of tomato diseases. After thorough analysis, they selected R-FCN with ResNet-50. This particular configuration achieved an impressive average precision (AP) of 85.98% and effectively recognized nine different diseases. In their study, Xu et al. (2022) presented a real-time technique for detecting diseases on cucumber leaves. In order to boost the model’s performance, channel pruning was utilized to trim and fine-tune a sparsely trained model, and the pruned YOLO v5s+Shuffle model was then deployed on the Jetson Nano platform, achieving a remarkable mean average precision (mAP) of 96.7%. Zhang et al. (2021) developed a multi-feature fusion Faster R-CNN to accurately detect diseases on soybean leaves. Their approach yielded a best average precision of 83.34%, showcasing the effectiveness of their design. Chen et al. (2022) developed an improved plant disease identification model based on the original YOLOv5 network model with an average accuracy of 70%. Roy et al. (2022) put forward an exceptional-performance framework for real-time detection of fine-grained objects. Their framework achieved successful detection of diseases across diverse and challenging environmental conditions.

Taking inspiration from attention mechanisms (Vaswani et al., 2017), some research studies have enhanced feature extraction by incorporating attention mechanisms. For example, Qi et al. (2022) put forward an enhanced network model, SE-YOLOv5 for the identification of tomato virus diseases, which resulted in an average precision (mAP) of 94.10%. In another study, Guo et al. (2022) presented a CST model based on the Swin Transformer. This model employed a novel convolution design and achieved accuracies of 0.909 and 0.922. Furthermore, Thai et al. (2023) introduced FormerLeaf. Their contribution was the proposal of attention pruning. This algorithm achieved a reduction in model size by 28%, an evaluation speed acceleration by 15%, and an approximate 3% improvement in accuracy.

Furthermore, the contextual information captured during the recording of plant disease images contributes to more accurate category classification by the model. Wang et al. (2020) introduced a context-aware attention model which encodes various types of information, such as image context, geographical information, time information, and environmental information, into image annotations. They utilized a multi-task learning architecture with CNN models for each task to extract features related to pest coarse classification, geography, time, and environment. This algorithm surpasses traditional image feature extraction by incorporating external environmental factors like geography, time, and environment into the process. By extracting features relevant to pest habitat, it explores the possibility of integrating a wide range of environmental information into CNN for feature representation. Zhao et al. (2020) developed the Multi-Context Fusion Network (MCFN), which leverages contextual features extracted from image sensors as prior knowledge. This introduction resulted in highly accurate predictions of crop diseases. Cheng et al. (2023) proposed a Position Attention Block that effectively extracts positional information from feature maps and constructs attention maps to bolster the feature extraction ability. These efforts aim to enhance the performance and applicability of disease object detection models in real-world agricultural settings.

### 2.4 Technical challenges of plant disease detection methods

**Figure 1:**
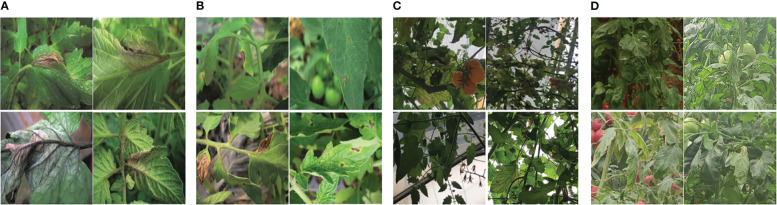
Various obstacles of plant disease detection task **(A)** intra-class discrepancies; **(B)** inter-class resemblances; **(C)** complications arising from low resolution; **(D)** occlusion overlap).

Compared to earlier studies on plant disease detection algorithms, the methods mentioned above have significantly improved detection performance. However, they still encounter various obstacles that pose challenges to accurate detection. These challenges include four categories (**Figure 1**). One of them is intra-class variation, where different instances of the same disease may exhibit variations in appearance and symptoms. Inter-class resemblances refer to cases where different diseases or healthy plants may share similar visual characteristics, leading to misclassification. Complications arising from low resolution images can make it difficult to discern fine details and accurately identify diseases. Additionally, occlusion and overlap of plant parts or other objects in the image can further hinder detection accuracy. It is crucial for researchers to address these challenges through advanced techniques such as data augmentation, model optimization, and incorporating contextual information to improve the robustness and reliability of plant disease detection algorithms (Thakur et al., 2022). **Figure 1** illustrates these challenges visually.

To tackle the aforementioned concerns, this study proposes a method for detecting objects related to diseases in tomato plants called PKAMMF. This method integrates a prior knowledge attention mechanism and incorporates features at different scales to tackle the obstacles of dense distribution of tomato disease objects in complex backgrounds, a broad spectrum of scale variations, and lack of feature information for small objects. By combining the prior knowledge attention mechanism and multi-scale features, PKAMMF aims to improve the performance of detecting tomato disease objects.

This study makes significant contributions in the following aspects:

(1) A backbone network was proposed, which integrates a prior knowledge attention mechanism to improve the capability of extracting features and improve model stability during training on large-scale datasets.
(2) The Rep Conv convolutional layer was reparameterized in a structured manner to construct the SPPCSPF module, reducing computational and memory costs during model training.
(3) A parallel multi-branch feature fusion network was established to minimize the loss of effective information in feature maps. Additionally, to enhance the capability of detecting small objects across multiple scales, an additional layer specifically designed for small object detection was incorporated.

(4) A novel A-SIOU loss function was employed to refine and improve bounding box regression, resulting in accelerated model convergence and improved training accuracy. Experiments were carried out to evaluate the effectiveness of the proposed approach using a self-built dataset of tomato disease. The findings indicate that the proposed method surpasses the performance of mainstream algorithms in tomato disease object detection tasks with complex backgrounds.

## 3 Methods

Because of the intricate background conditions of tomato disease images, where the background occupies a large portion of the image while the diseased area to be detected is often small, it is necessary to use a network structure with the ability to globally model the complex nature of the background. Therefore, this study proposes a tomato disease object detection method that combines prior knowledge attention mechanism and multi-scale features. The specific improvements are described as follows:

### 3.1 Prior knowledge attention mechanism module

**Figure 2:**
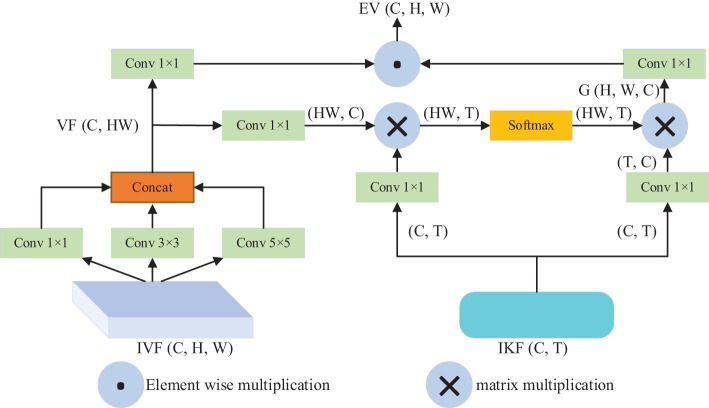
Framework of PKAM module.

To improve disease detection and recognition, it is necessary to incorporate geographical location information, environmental parameters, and time information of tomato disease images. This is due to the inconsistent types of tomato diseases, occurrence time, surrounding environment, and geographical conditions. In this study, we propose a PKAM module that integrates the prior knowledge attention mechanism to enhance the capability of extracting target features in complex backgrounds. The framework of the PKAM module is illustrated in **Figure 2**.

Firstly, to encode the prior knowledge of tomato disease, we utilize the Bert model (Devlin et al., 2018). The Bert model, introduced by Google in 2018, is a language model built on a transformer encoder structure. It is specifically designed for encoding language information. In our case, the Bert model is employed to encode the prior knowledge of tomato diseases. By inputting the prior knowledge text information, the model generates an encoded prior knowledge vector K (C, T), where C denotes the maximum text length of the prior knowledge and T denotes the vector dimension. The default value for T is set at 100.

Furthermore, to handle the diverse perspectives of tomato disease images, we utilize convolutional kernels of various sizes (1×1, 3×3, 5×5) to extract features from the input visual features IVF (C, H, W). These convolutional kernels capture different spatial information at different scales. Afterwards, the features obtained at different scales are concatenated and merged to obtain the visual feature VF (CH, H, W). Additionally, we treat the obtained visual feature VF as the query Q, the input knowledge feature IKF as the key K and value V, and employ scaled dot-product attention to calculate the output feature map G (H, W, C). The calculation formula for this attention mechanism can be expressed as follows:


(1) $$Attention\left(Q,K,V\right)=Softmax\left(\frac{Q,K^{T}}{\sqrt{d}}\right)V$$

In the above-mentioned formular, d denotes the dimension of the vectors Q and K.

Then, the fundamental concept behind the prior knowledge embedding strategy is to integrate visual information with prior knowledge through the attention mechanism. By combining the encoded knowledge features with the visual features corresponding to the input image, we achieve enhanced visual features that incorporate prior knowledge about tomato diseases. The output feature map G is multiplied by the visual features VF, resulting in the final enhanced visual features E-V (C, H, W) that are embedded with prior knowledge about tomato diseases.

In the end, the advantageous enhanced visual features EV resulting from the fusion process will be learned by the PKAM module in the subsequent encoding stage, thereby producing an output vector embedded with prior knowledge of tomato diseases. Here, C and C_1_ represent the channel count in the feature maps, while H and W denote the height and width of the feature maps, respectively.

### 3.2 SPPCSPF module with structural reparameterization

This research has developed the SPPCSPF module to reduce memory access costs and improve model training efficiency. The module employs structurally reparameterized RepConv convolutional layers in place of regular convolutional layers within the residual structure. In training, a multi-branch residual structure is used for feature extraction. Additionally, during inference, the convolutional layers are merged with BN (Batch Normalization) layers, and the three branches are consolidated into a single-path model. As a result, all the trained parameters are equivalent to a 3×3 convolutional layer, enabling faster inference speed and reduced memory access costs.

**Figure 3:**
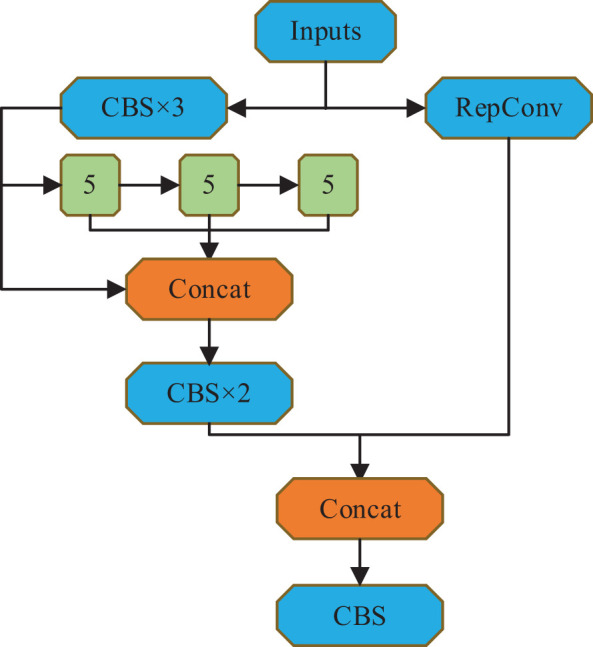
Network structure of SPPCSPF module.

**Figure 3** illustrates the designed max pooling part of the SPPCSPF module, taking into consideration the increased training costs associated with using structurally reparameterized layers. The pooling kernel size is set to a fixed value of 5×5 in this design. Furthermore, the output from each pooling layer serves as input for the subsequent pooling layer. By setting a pooling stride of 1, two 5×5 max pooling layers can effectively perform the same function as a single 9×9 max pooling layer. Similarly, three 5×5 max pooling layers yield the same result as a single 13×13 max pooling layer. This approach significantly reduces the computational load during model training.

### 3.3 Multiscale detection module with small object detection layer

In this study, the target detection dataset images are of size 800×800. Since there are small objects with pixel sizes smaller than 8×8 in tomato disease detection images that have complex backgrounds, the study employs the principle of detecting large objects based on the small object detection feature map and vice versa. To maintain the same scale of the output feature map as the baseline model, a 160×160 detection layer is added in the prediction stage. This layer divides the input image into 160×160 grid cells, each measuring 5×5 in size. This approach enhances the regression and adjustment of prior boxes, resulting in accurate detection boxes for small objects and significantly enhancing precision in small object detection. To tackle the challenge of losing a substantial amount of feature information for smaller objects in deeper network layers, this study proposes the incorporation of innovative feature layers with alternative dimensions sourced from the backbone network. Furthermore, by enhancing the Neck component and constructing a parallel multi-branch feature fusion network, the loss of effective information in the feature maps is mitigated.

### 3.4 A-SIOU loss function

Precise target localization is essential for successful target detection, relying heavily on the utilization of a superior quality bounding box loss function. The conventional CIOU loss function proficiently handles the task of orienting bounding boxes, even in situations where there is no intersection between the predicted and ground truth boxes. Accomplishing this involves incorporating the aspect ratio of the boxes into the loss calculation. However, the CIOU loss function has certain limitations, as it calculates all loss variables as a whole without adequately addressing the disparity between the actual target and the predicted box. As a result, this approach leads to slow convergence and instability issues.

In this study, we introduce the A-SIOU (Alpha SIOU) loss function as a replacement for the existing CIOU loss function in the tomato disease detection model. This novel bounding box loss function, based on enhancements to the SIOU loss function (Gevorgyan, 2205), offers significant improvements. It enhances the gradient convergence speed of the loss function through the parameter α (He et al., 2021). The A-SIOU loss function fully considers the influence of distance, angle, and area - these three key factors - on the boundary regression of the model. This ensures that the predicted box can converge towards the ground truth box more quickly, thereby controlling the convergence direction. The proposed loss function consists of four components: L_angle_, L_dis_, L_shape_, and IOU.

The equation for computing the angle loss L_angle_ is given by:


(2) $$\{\begin{array}{c} z_{h}=\left|b_{cy}^{t}-b_{cy}^{p}\right|\\ \sigma =\sqrt{{\left(b_{cx}^{t}-b_{cx}^{p}\right)}^{2}{\left(b_{cy}^{t}-b_{cy}^{p}\right)}^{2}}\\ L_{angle}=1-2\sin^{2}\left(arcsin\left(\frac{z_{h}}{\sigma }\right)-\frac{\pi }{4}\right) \end{array}$$

In the given equation, *z_h_* denotes the disparity in height between the center points of the predicted box and the ground truth box. *σ* represents the spatial displacement between the center coordinates of the ground truth box and the predicted box. Furthermore, $b_{cx}^{t}$ and $b_{cy}^{t}$ denote the center coordinates of the ground truth box, while $b_{cx}^{p}$ and $b_{cy}^{p}$ denote the center coordinates of the predicted box.

The formula for calculating the distance loss is provided below:


(3) $$\{\begin{array}{c} \rho_{x}={\left(\frac{b_{cx}^{t}-b_{cx}^{p}}{c_{w}}\right)}^{2}\\ \rho_{y}={\left(\frac{b_{cy}^{t}-b_{cy}^{p}}{c_{h}}\right)}^{2}\\ L_{dis}=2-\sum_{i=x,y}exp\left(\left(L_{angle}-2\right)\rho_{i}\right) \end{array}$$

Here, *c_w_* and *c_h_* represent the width and height of the minimum bounding rectangle of the ground truth box and the predicted box.

To compute the shape loss, use the following formula:


(4) $$\{\begin{array}{c} \omega_{\omega }=\frac{\left|\omega^{p}-\omega^{t}\right|}{max\left(\omega^{p},\omega^{t}\right)}\\ \omega_{h}=\frac{\left|h^{p}-h^{t}\right|}{max\left(h^{p},h^{t}\right)}\\ L_{shape}=\sum_{i=w,h}{\left(1-exp\left(\omega_{i}\right)\right)}^{\theta } \end{array}$$

In the equation above, *ω^p^* and *h^p^* represent the width and height of the predicted box, while *ω^t^* and *h^t^* represent the width and height of the ground truth box. *θ* is a constant used to control the emphasis on shape loss, with a value of 4 in this study.

The formula for calculating the A-SIOU loss function is as follows:


(5) $$\{\begin{array}{c} IOU=\frac{A}{B}\\ L_{A-SIOU}=1-\left(IOU^{\alpha }-\frac{\left(L_{dis}{\;}^{\alpha }+L_{shape}{\;}^{\alpha }\right)}{2}\right) \end{array}$$

In the equation above, A and B represent the intersection and union of the areas of the ground truth box and the predicted box. *α* is a variable that controls the convergence speed of the loss function. Through multiple experiments, it has been found that setting *α* to 2 helps the model focus more on targets with high intersection-over-union ratios, thereby improving the accuracy of object localization.

Compared to other functions, the A-SIOU boundary box loss function considers the influence of distance and angle on boundary regression, thereby avoiding the issue of gradient vanishing in cases where there is no overlap between the predicted box and the ground truth box. Additionally, the A-SIOU loss function include four components. In the angle loss component, the range of values for the angle loss *L_angle_* is [0, 1] due to the characteristics of the sine function. In the distance loss component, considering the range of *ρ_i_* to be [0, 1), the value range of the distance loss *L_dis_* can be derived as (0, 2-2e-2). In the shape loss component, considering the range of *ω_i_* to be (0, 1), the value range of the shape loss *L_shape_* can be derived as (0,2(1-e)^4^). In conclusion, the A-SIOU function has a value range of (-2 + 2e^-4^,1 + 2(1-e)^8^), which has both upper and lower limits, effectively preventing gradient explosion.

### 3.5 Overall framework of PKAMMF

**Figure 4:**
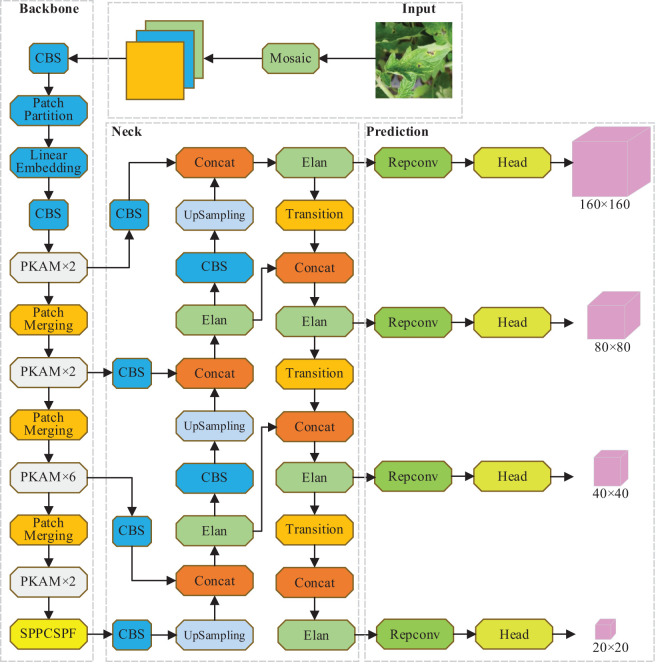
Overall framework of PKAMMF.

Based on the above improvement measures, the overall network framework of the PKAMMF method for tomato disease detection, which incorporates the fusion of prior knowledge attention mechanism and multi-scale features to enhance performance, is illustrated in **Figure 4**.

## 4 Dataset

### 4.1 Experimental data collection

**Figure 5:**
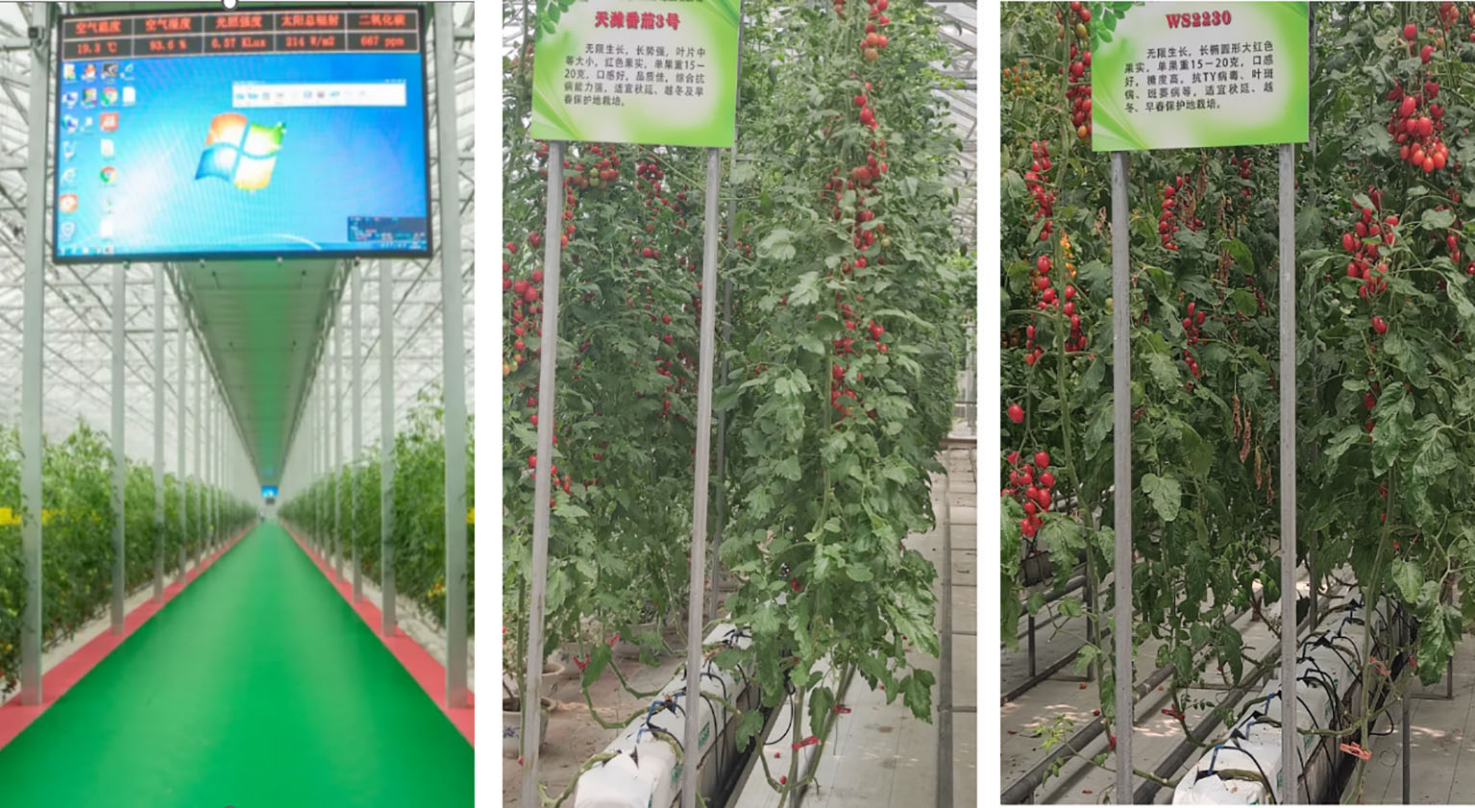
Experimental site.

The experimental research area was selected as the tomato planting base in Shouguang City, Shandong Province. This location is known for year-round cultivation of various tomato varieties, as shown in **Figure 5**. To collect data, agricultural IoT monitoring equipment equipped with a 4K high-definition camera (with a resolution of 4096x3112) was used. The equipment enabled the collection of typical tomato disease images from different plants, regions, and growth stages under natural conditions. Image collection took place during specific time intervals: 08:00 to 09:00, 11:00 to 12:00, and 15:00 to 16:00 every day, to capture images under varying lighting conditions. In total, 26,983 images depicting various types of tomato diseases were collected.

### 4.2 Dataset construction

To ensure data quality, the original image was cropped to a fixed size of 640 × 640. Cropping the image to this specific dimension ensures that the subject of the photograph is clear, the disease objects are easily discernible, and the real background is visible. In addition, we manually removed duplicate and low-quality images from the dataset. After the selection process, we obtained a total of 10,000 tomato disease images that represent various types of diseases. Next, we divided these images into a 9:1 ratio, creating a training set and a test set. The training set encompassed 9,000 images, while the test set contained 1,000 images. By including a diverse range of scene information, such as rainy and foggy weather, sunny days, cloudy conditions, and other scenarios, the dataset effectively captures real-world planting environment information.

In order to enhance the model’s robustness to variations in tomato disease image sizes and lighting conditions, we employed a method to augment the training set. This involved changing the contrast and scaling the image sizes. The contrast coefficient and scaling factor were randomly generated within the ranges of [0.6, 1.5] and [0.6, 1.7], respectively. In order to enhance the model’s capability in detecting occluded disease objects, we augmented the dataset by adding salt-and-pepper noise to simulate random pixels and artificially create occlusions. As a result, the augmented training set consisted of a total of 45,000 tomato disease images. Meanwhile, the test set remained in its original state, and data augmentation was solely applied to the training set. This decision was made to improve the dependability and precision of the test results.

### 4.3 Data annotations

The labeling of tomato disease samples is divided into two steps. Firstly, the prior knowledge information of tomato disease is labeled. Secondly, the tomato disease category information is labeled.

#### 4.3.1 Labeling prior knowledge information of tomato diseases

The prior knowledge information of tomato diseases includes the identification of the disease infection location (leaves, stems, fruits) and the shooting angle from which the images are captured (main view, top view). To illustrate this, **Table 1** presents a compilation of tomato disease images along with corresponding label examples.

**Table 1:** Tomato disease images and label examples.

Image No.	1	2	3	4
Tomato disease image	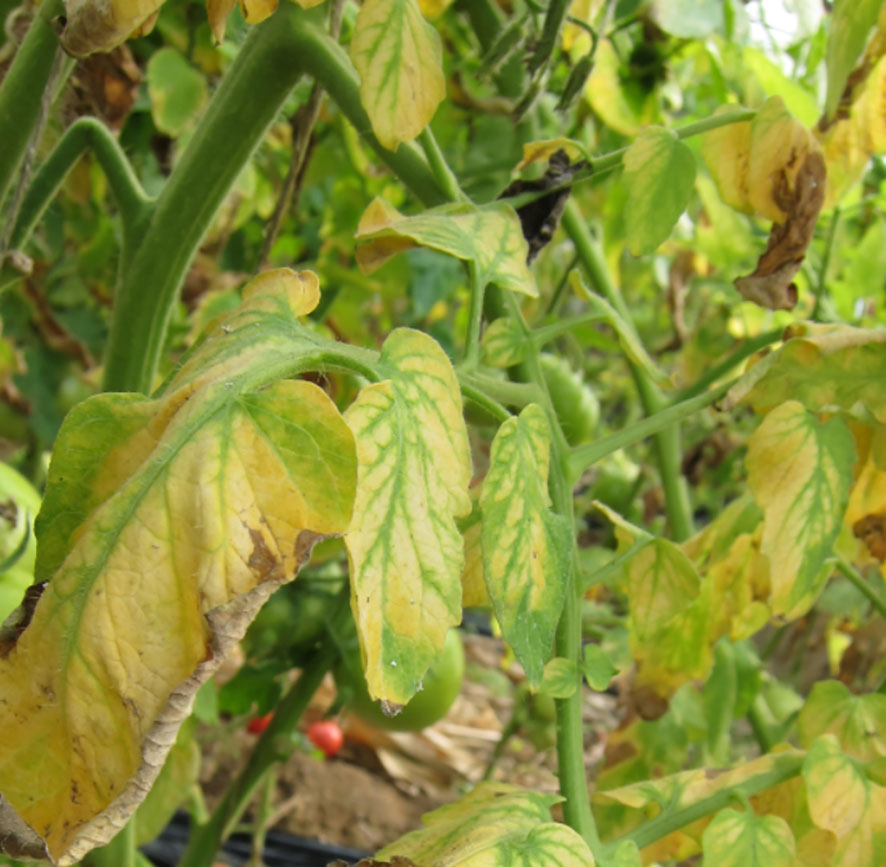	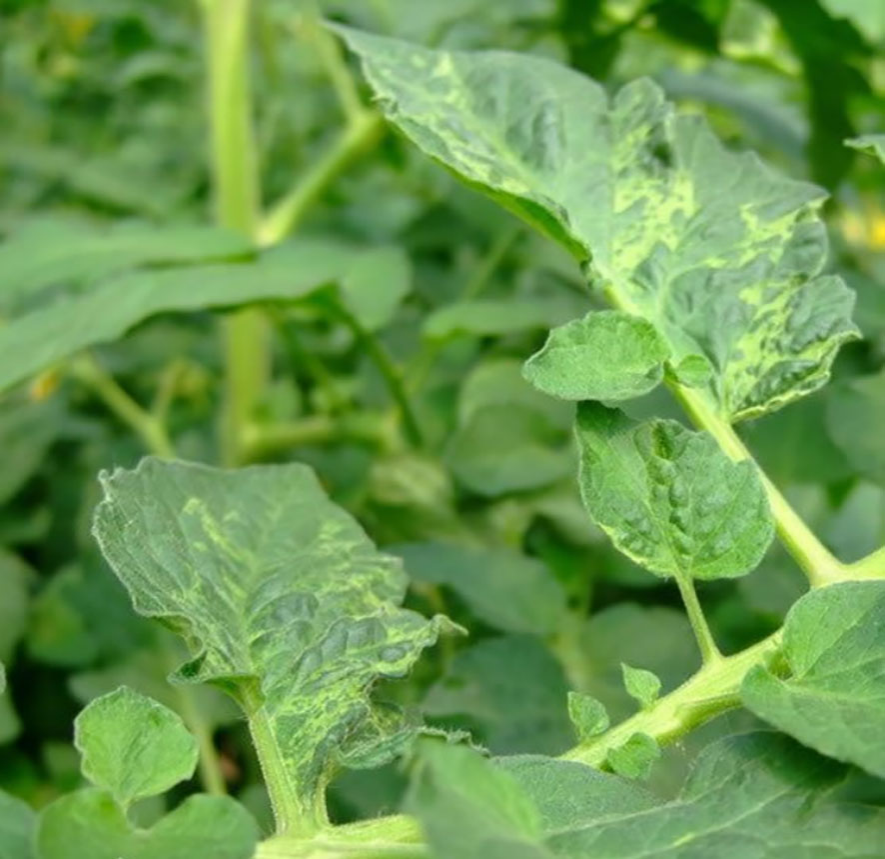	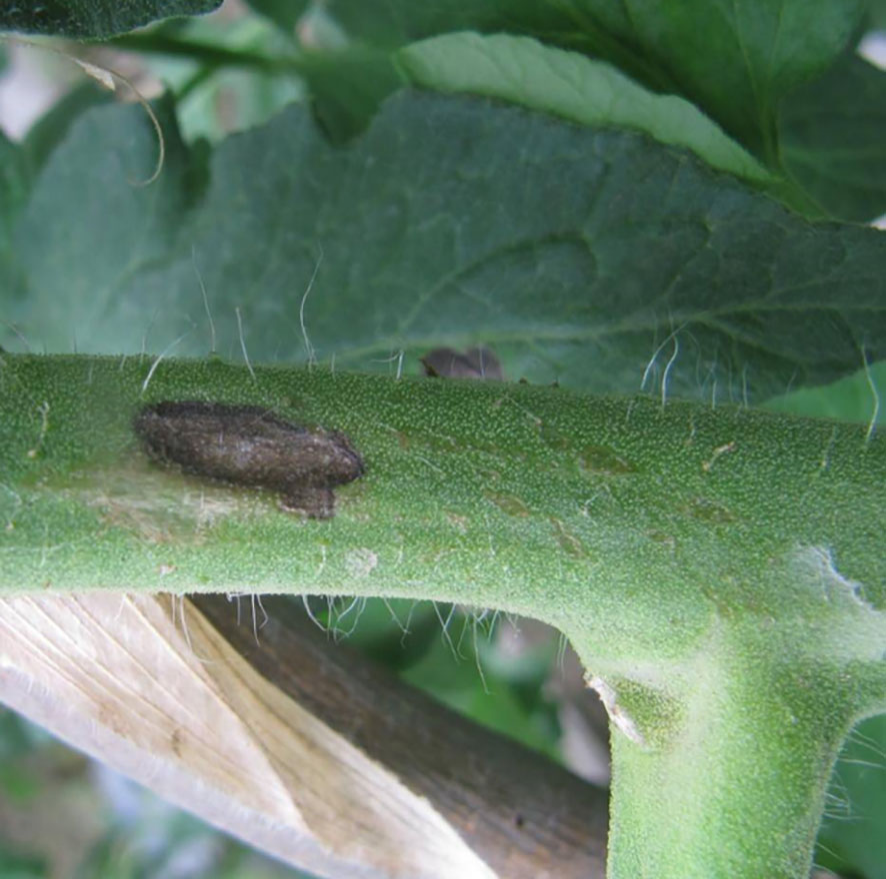	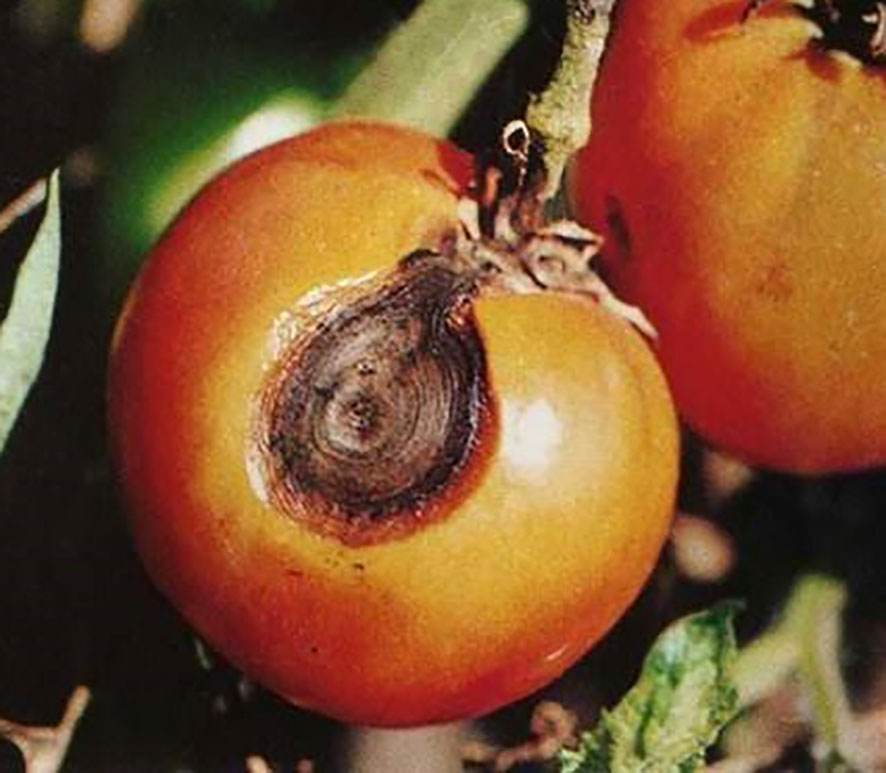
Location	Leaves	Leaves	Stems	Fruits
Shooting Angle	Main View	Top View	Top View	Top View
Label	Pk-lm	Pk-lv	Pk-sv	Pk-fm

The labeled examples serve as valuable references for training and developing models or systems focused on tomato disease detection, making use of prior knowledge information.

#### 4.3.2 Labeling tomato disease category information

The model used in this study was trained on a Pascal VOC-formatted dataset. To annotate the tomato disease images of different disease types, we utilized the LabelImg software. The annotation rules were as follows: 1) We annotated the diseased areas in the images without occlusion or with some occlusion that did not impact manual judgment of the disease type. 2) We did not annotate severely occluded areas where it was difficult for humans to determine the disease type accurately. Since tomato diseases rapidly spread, it is common for most images to contain multiple affected areas. In total, we annotated 127,356 diseased areas across 10 disease categories during the annotation process. The quantities of the different tomato disease types can be found in **Table 2**.

**Table 2:** Sample quantities for each disease type.

No.	Disease class	Sample images in the training set	Annotated diseased areas in the training set	Sample images in the test set	Annotated diseased areas in the test set
1	Early blight	4500	10903	100	228
2	Late blight	4500	12317	100	247
3	Bacterial spot	4500	17302	100	469
4	Gray leaf spot	4500	13236	100	303
5	Gray mold	4500	12315	100	269
6	Leaf mold	4500	11871	100	235
7	Yellow leaf curl virus	4500	10036	100	213
8	Mosaic virus	4500	10277	100	208
9	Canker	4500	12398	100	249
10	Anthracnose	4500	13964	100	316
	Total	45000	124619	1000	2737

## 5 Results

### 5.1 Operating environment configuration

The experimental platform employs Ubuntu 22.04 as its operating system. It utilizes two NVIDIA RTX 3080 GPUs for deep learning, with a memory capacity of 12GB. Other software packages include Python 3.8, CUDA 11.0, Torch 1.7.0, and torchvision 0.8.1.

### 5.2 Model training

During the training phase, we performed pre-training using the weight file of the baseline model. Since the improved model shares most of its structure with the baseline model, many weights can be transferred from the baseline model to the improved network. This transfer of weights allows us to save a significant amount of training time. To carry out the training, a batch size of 16 was chosen, and the training process consisted of 300 epochs. We utilized the Adam algorithm for gradient descent. Additionally, the image size was adjusted to 416×416. The initial learning rate was set to 0.01, and the weight decay coefficient was determined to 0.000005. We employed the cosine annealing algorithm for learning rate adjustment.

**Figure 6:**
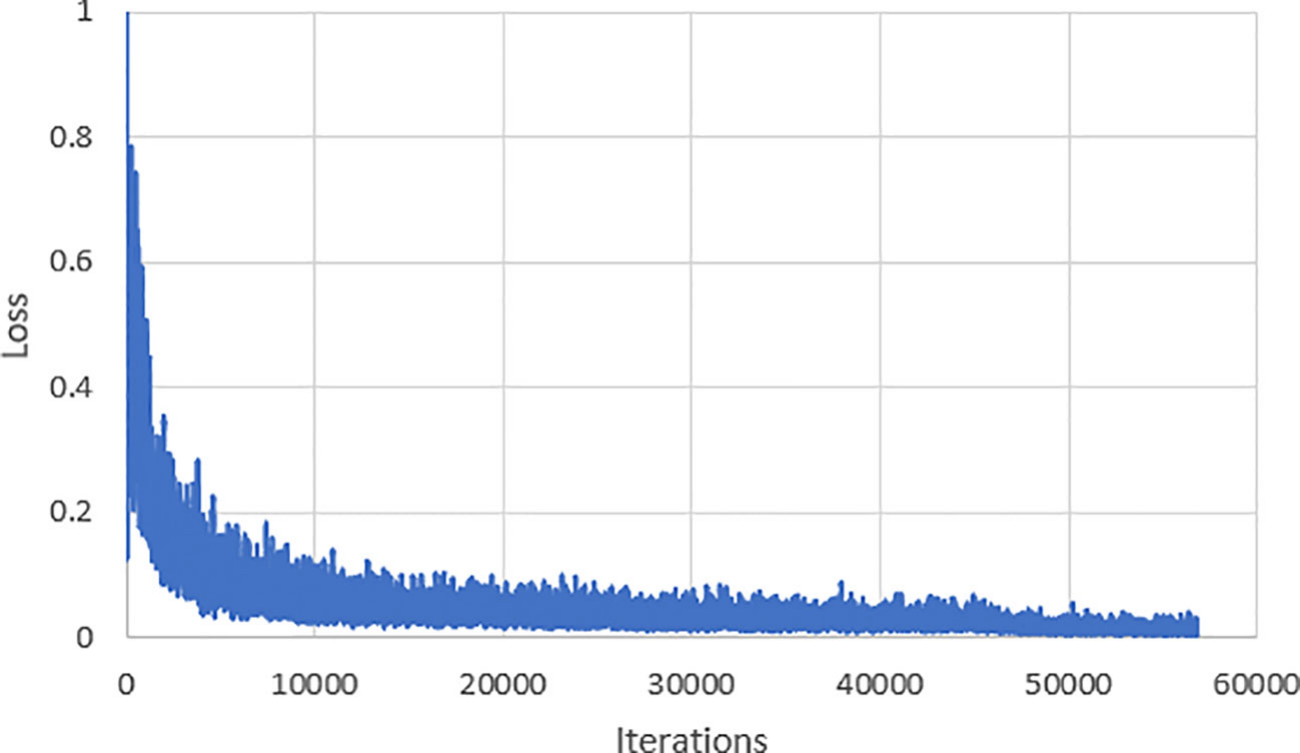
Variations of the loss function during the training process.

Throughout the process of training the model, we recorded the loss function of the model and depicted it in **Figure 6**. According to the depicted graph, in the early stages of training, the loss function experiences rapid decrease with minor overall fluctuations. Notably, around the 20,000th iteration, the loss value reaches 0.022. After training for 30,000 iterations, the loss value converges and stabilizes at 0.016.

### 5.3 Metrics for evaluating performance

Before introducing the metrics, it is necessary to briefly explain the symbols used. In this research experiment, when IOU > 0.5, it is considered that the predicted box hits the annotated box; otherwise, it is considered that the predicted box does not hit the annotated box. TP indicates the count of correctly predicted boxes that match the annotated boxes for the given class, FP corresponds to the count of incorrectly predicted boxes that match the annotated boxes for the given class, TN is the count of predicted boxes that correctly match the annotated boxes for other classes, and FN represents the count of predicted boxes that fail to match any annotated boxes.

The commonly used metrics include Recall, Precision, Average Precision (AP), and mAP. Recall is used to evaluate whether the model predicts all target objects comprehensively. The model’s prediction accuracy is assessed through Precision. To evaluate the model’s classification performance for a particular class, AP calculates the area under the Precision-Recall curve, while mAP computes the average AP across multiple classes. Furthermore, the model’s detection speed is measured in frames per second (FPS), representing the number of images detected per second. The formulas for calculating the above evaluation metrics are as follows:


(6) $$Recall=\frac{TP}{TP+FN}$$

(7) $$Precision=\frac{TP}{TP+FP}$$

(8) $$AP=\int_{0}^{1}p\left(r\right)dr$$

(9) $$mAP=\frac{\sum_{n=1}^{N}AP\left(n\right)}{N}$$

(10) $$FPS=\frac{C_{img}}{Time_{detect}}$$

In the above-mentioned formulars, *p*(r) represents the Precision-Recall curve. *N* denotes the overall count of categories within the tomato disease detection data, while *n* represents the current data category. *C_img_* represents the count of pictures within the test dataset, and *Time_detect_* represents the time taken to detect *C_img_* images.

### 5.4 Ablation experiment

In order to verify the performance enhancement of the various improvement measures proposed in this study for tomato disease image object detection, a series of experiments involving ablation was conducted. These experiments aimed to systematically assess the impact of each improvement measure by selectively removing or disabling them one by one. The experimental results are presented in **Table 3**, where PKAM, SPPCSPF, MSD, and A-SIOU represent the four improvement measures, namely, the PKAM module, the SPPCSPF module, the Multi-Scale Detection module, and the loss function, respectively.

**Table 3:** Results of ablation experiments.

Methods	PKAM	SPPCSPF	MSD	A-SIOU	Params(M)	FPS(Frames per second)	mAP(%)
**YOLOv7**					36.853	66.39	88.10
**Improvement 1**	**Yes**				50.856	57.92	90.33
**Improvement 2**	**Yes**	**Yes**			51.267	57.98	90.92
**Improvement 3**	**Yes**		**Yes**		51.698	55.67	91.07
**Improvement 4**	**Yes**	**Yes**	**Yes**		51.279	55.32	91.37
**PKAMMF**	**Yes**	**Yes**	**Yes**	**Yes**	51.723	54.68	91.96

The above-mentioned table reveals the following:

(1) Improvement 1 designs the PKAM module, which utilizes attention mechanisms to integrate visual information with prior knowledge. This enhances the network’s ability to capture global observations. While the introduction of the PKAM module leads to a slight increase in the parameter count and a decrease in detection speed, it results in a significant improvement in mAP, with an increase of 2.23%. This indicates that the incorporation of the PKAM module helps to improve the overall performance of the detection by effectively leveraging both visual information and prior knowledge. Despite the trade-off in terms of computation and speed, the gained improvement in accuracy justifies the utilization of the PKAM module in the context of disease object detection in tomato plants.
(2) Building upon improvement 1, improvement 2 designs the SPPCSPF structure with structurally reparameterized RepConv convolutional layers. This design not only stabilizes the training process but also improves inference speed, resulting in an mAP improvement of 0.59% compared to improvement 1.
(3) Based on improvement 1, improvement 3 implements multi-scale detection by adding a new feature fusion layer in the Neck section and incorporating a small object prediction layer during inference. Compared to improvement 1, improvement 3 achieves a mAP improvement of 0.74%, indicating enhanced accuracy in detecting small objects.
(4) Improvement 4 integrates the first three improvements and achieves the highest mAP. Compared to the baseline model, improvement 4 shows an mAP enhancement of 3.86%.
(5) Continuing from improvement 4, this study further improves the proposed method by utilizing A-SIoU. Although the mAP improvement is only 0.59% compared to improvement 4, the convergence speed during actual training is faster.

Overall, through multiple improvement measures, the proposed method in this study achieves a 3.86% mAP increase compared to the baseline model. Despite a slight increase in parameter count, the number of parameters remains within the same order of magnitude as the baseline model, indicating that the additional computational requirements are manageable. The detection frame rate drops by 11.71 frames per second. However, it still meets the fundamental criteria for real-time performance. Considering these factors, the detection accuracy improvement obtained through the various improvement measures is highly cost-effective. The trade-offs in terms of parameter count and detection speed are reasonable, given the substantial enhancement in accuracy achieved by the proposed method. This implies that the proposed method may have practical value and can be considered as an effective solution for detecting objects related to diseases in tomato plants.

### 5.5 Comparative analysis of performance with alternative mainstream approaches

To further validate the advantages of the PKAMMF method in detecting tomato diseases in complex backgrounds, we conducted object detection experiments using our own tomato disease dataset under the same experimental environment and parameter settings. We compared and analyzed the object detection performance of mainstream methods such as Faster-RCNN, SSD, YOLO series, and PKAMMF in this study. The experimental outcomes from these various methodologies are presented in **Table 4**.

**Table 4:** Performance metrics of various methods.

Methods	P(%)	R(%)	F_1_ score(%)	Params(M)	FPS	mAP(%)
Faster-RCNN	85.98	61.79	71.85	129.8	10.59	70.73
SSD	90.87	55.87	68.92	25.43	47.65	72.54
YOLOv3	85.88	60.78	70.82	60.83	27.28	78.62
YOLOv4	87.95	68.54	76.21	62.73	34.07	80.37
YOLOv5	89.97	75.82	80.33	70.10	60.28	88.98
YOLOv7	88.64	83.52	85.97	36.853	66.39	88.10
PKAMMF	91.90	86.07	88.18	51.723	54.68	91.96

As shown in **Table 4**, the parameter size (Params) of single-stage object detection methods, including SSD, YOLO series, and the proposed method in this study, is relatively smaller than the two-stage object detection approach, Faster-RCNN. This reduction in parameter size leads to faster detection speed. In comparison to the baseline model, the proposed method in this study exhibits an increase of 14.87M parameters and a decrease of 11.71 frames per second (FPS) in the detection frame rate. However, it demonstrates an improvement of 3.26% in precision (P), 2.55% in recall (R), and achieves a mean average precision (mAP) of 91.96%. This performance enhancement surpasses YOLOv7 by 3.86% and outperforms other models in the table. It strongly indicates that PKAMMF exhibits remarkable superiority in detecting tomato disease objects.

Given that the self-built tomato disease dataset comprises ten disease classes with significant variations in scale and features among different instances, achieving multi-scale object detection is typically challenging. **Table 5** presents the average precision (AP) results of different methods for detecting various object categories.

**Table 5:** Average precision of several methods for detecting different disease class.

Disease class	Faster-RCNN	SSD	YOLOv3	YOLOv4	YOLOv5	YOLOv7	PKAMMF
**Early blight**	89.3	90.2	90.6	90.1	95.4	96.7	98.3
**Late blight**	88.7	89.3	92.5	80.3	91.3	92.2	97.9
**Bacterial spot**	77.5	70.2	79.8	80.6	76.3	78.5	79.6
**Gray leaf spot**	77.3	80.1	72.7	75.8	76.2	76.4	79.1
**Gray mold**	69.4	66.8	92.1	88.9	90.0	90.2	93.4
**Leaf mold**	89.3	80.4	80.8	85.4	88.6	89.7	94.8
**Yellow leaf curl virus**	86.5	84.3	78.6	79.6	82.7	84.9	90.1
**Mosaic virus**	80.2	86.7	82.4	72.8	87.9	88.1	89.6
**Canker**	53.7	52.2	69.7	70.7	88.2	90.1	90.3
**Anthracnose**	42.6	53.9	80.4	76.9	89.3	90.5	91.1

According to **Table 5**, the experiment shows that the proposed PKAMMF outperforms the baseline model YOLOv7 among the 10 target categories. Therefore, in comparison to alternative prevalent object detection methods, PKAMMF also demonstrates significant advantages in detecting objects at multiple scales. The proposed method exhibits notable superiority in detecting tomato disease objects of varying scales, even within complex backgrounds. However, there is still room for improvement in detecting bacterial spot disease and gray leaf spot disease, as these targets have less distinct features. In scenarios involving multiple scales, there is a higher risk of false negatives occurring.

### 5.6 Performance comparison of different attention mechanisms in tomato disease detection

The comparative experiments were conducted under consistent conditions, and several classical attention mechanisms, namely SE (Hu et al., 2018) (Squeeze and Excitation), CBAM (Woo et al., 2018) (Convolutional Block Attention Module), GAM (Liu et al., 2021) (Global Attention Mechanism), and Biformer (Zhu et al., 2023), were added. **Table 6** provides a performance comparison of the proposed prior knowledge attention mechanism and other attention mechanisms in tomato disease detection. The results clearly demonstrate that the proposed prior knowledge attention algorithm in this study achieves the highest mAP and exhibits a significant enhancement in detection performance.

**Table 6:** Comparison of different attention mechanisms.

Algorithms with different attention mechanisms	mAP(%)
baseline	88.10
baseline+SE	88.26
baseline+ CBAM	89.39
baseline+ GAM	89.27
baseline+Biformer	89.56
baseline+PKAM	90.33

This finding suggests that the prior knowledge attention mechanism effectively integrates prior knowledge of tomato diseases, enabling more focused feature extraction in the regions associated with tomato diseases. The prior knowledge attention mechanism helps to focus on relevant areas of the image that are more likely to contain disease objects. This can improve the efficiency and accuracy of detection by reducing false positives and identifying subtle disease symptoms. Consequently, the prior knowledge attention mechanism is deemed more suitable for feature extraction in tomato disease detection scenarios.

### 5.7 The influence of training samples of different sizes on detection results

**Figure 7:**
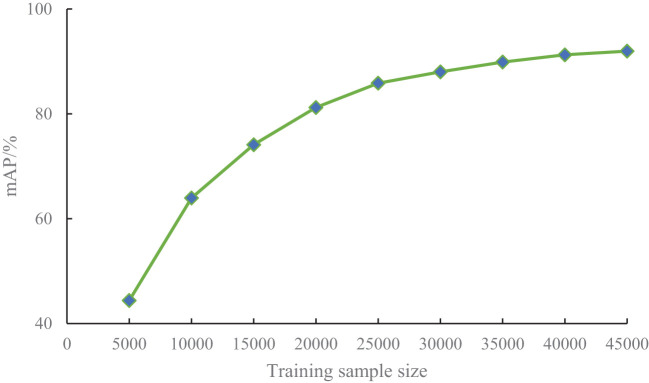
The impact of training sample size on detection results.

The number of training samples can significantly influence the detection performance of a model, so training samples of different sizes should be evaluated. To investigate the impact of training sample size on the performance of the proposed PKAMMF model, it is necessary to employ a method that involves changing the number of training samples while keeping the test set samples unchanged. Building upon the experiments conducted in the previous sections, we randomly selected 5000, 10000, 15000, 20000, 25000, 30000, 35000, 40000, and 45000 samples, maintaining the same proportion of tomato disease categories from our self-built dataset. We employed the same training method to train datasets with varying sample sizes. The impact of training samples of different sizes on mean Average Precision (mAP) is illustrated in **Figure 7**.

From **Figure 7**, it can be seen that the learning ability of the model increases with the increase of sample size for different training sample Quantities. In the case of a small number of training samples (5000-200000), the mAP obtained in the experiment significantly can improve greatly. As the number of training samples continue to increase, the improvement of mAP slows down. When the number of training samples exceeds 30000, mAP gradually tends to stabilize.

### 5.8 Analysis of tomato disease object detection results

The proposed PKAMMF model was utilized to detect 10 types of tomato disease images under complex backgrounds in the test set, which comprised 1000 images. **Figure 6** presents some of the disease detection results.

**Figure 8:**
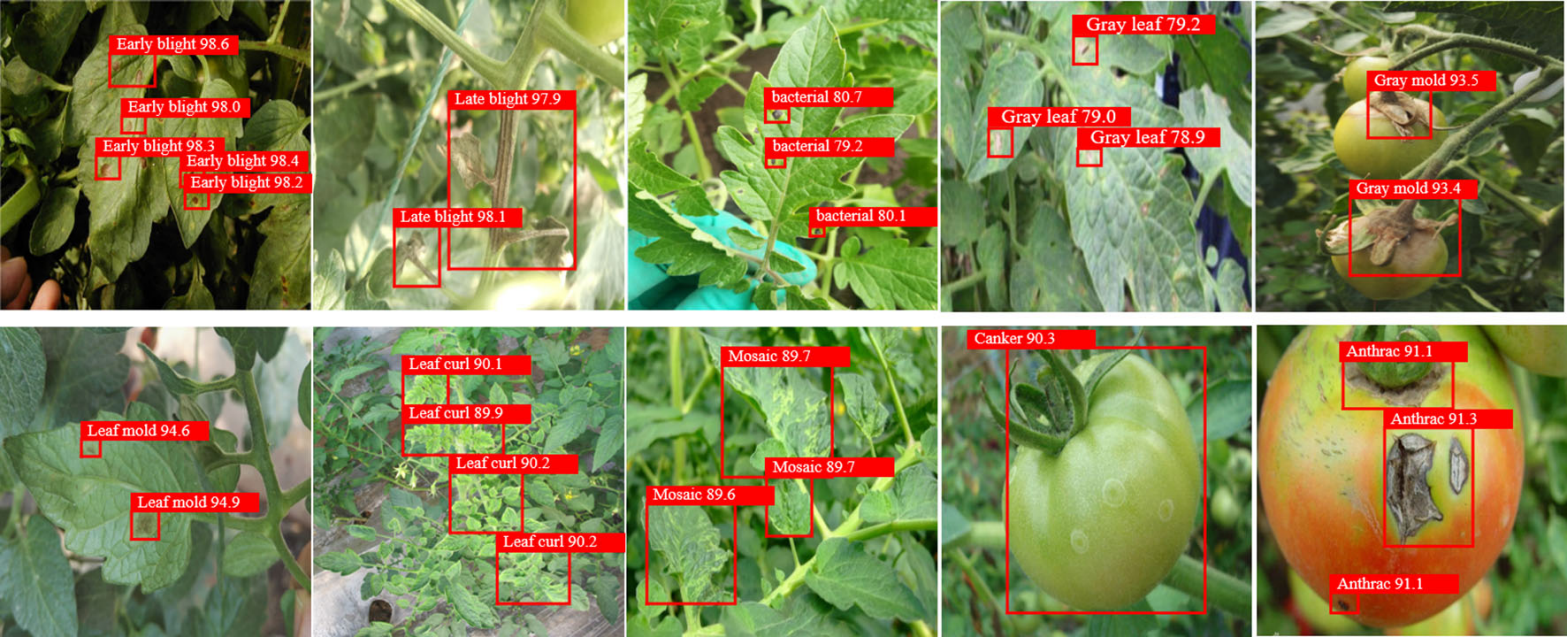
Object detection results of tomato disease.

Based on **Figure 8**, it is evident that the proposed PKAMMF model exhibits accurate detection capabilities for tomato disease images. The results depicted in **Figure 6** clearly showcase the robustness and adaptability of the PKAMMF model in handling challenging scenarios commonly encountered in tomato disease detection. The model effectively addresses the difficulties posed by objects at different scales, instances where objects are partially obscured, and varying lighting conditions, all of which are prevalent in real-world situations. Also, by effectively identifying objects in tomato disease images under challenging conditions, the model minimizes instances where diseases go undetected (missed detections) and also reduces the occurrence of incorrectly identifying healthy regions as diseased (false detections). By accurately detecting objects under such conditions, the PKAMMF model proves its capability to improve the performance of tomato disease detection. Its ability to handle complex backgrounds further strengthens its practical applicability in agricultural settings.

## 6 Discussion

In response to the limitations of existing deep learning models in learning prior knowledge of tomato disease objects and their reliance solely on visual features, this study proposes a method for tomato disease detection. The main objective is to leverage the prior knowledge available in tomato disease images and achieve accurate disease detection in complex backgrounds. To address this challenge, our proposed method, called tomato disease object detection method combining Prior Knowledge Attention Mechanism and Multiscale Features (PKAMMF), is introduced. By integrating the visual features extracted from detected images with the prior knowledge of tomato diseases, the overall performance of tomato disease detection in complex natural backgrounds is significantly enhanced. Through comprehensive experimental analysis and comparisons with existing methods, we have drawn the following discussions:

(1) In response to the challenge posed by complex backgrounds and unclear, overlapping target features in tomato disease images, this study investigates the utilization of prior knowledge on tomato diseases. We incorporate prior knowledge as auxiliary information into our model, enabling the detection network to effectively learn the distinctive features of various categories of tomato diseases and achieve accurate detection.
(2) Incorporate a feature fusion layer within the Neck section to facilitate effective information transmission across the backbone network. Additionally, augment the prediction section with a small object detection layer, enabling improved performance in detecting small objects at multiple scales. This enhancement reduces both the missed detection rate and false detection rate. Moreover, introduce the A-SIoU loss function to expedite bounding box regression, thereby accelerating the convergence speed.
(3) Validate the proposed algorithm using a self-built dataset specifically designed for tomato disease detection. The experimental results demonstrate that the proposed model adeptly utilizes the prior knowledge inherent in tomato disease images. It achieves accurate detection of small target diseases and effectively identifies densely occluded diseases against complex backgrounds. This approach significantly enhances the overall detection performance of tomato diseases and mitigates the occurrence of missed and false detections arising from complex backgrounds. Furthermore, the proposed model exhibits good real-time performance.

This study focuses on leveraging prior knowledge to enhance the detection effectiveness of tomato diseases. The experimental results validate that, with the guidance of prior knowledge, the model performs significantly better in detecting tomato diseases amidst complex natural backgrounds. This research sets the groundwork for integrating prior knowledge of tomato diseases with deep learning models, offering new insights and ideas for intelligent disease detection technology in plants. However, it is important to note that the proposed model currently only incorporates explicit knowledge, such as the precise location and shooting angle of tomato diseases, at the coding level. It lacks the capability to autonomously acquire implicit knowledge, including expert experience and the utilization of existing “knowledge” for reasoning. Therefore, future work should explore ways to integrate implicit knowledge, such as expert experience, into the model by employing technologies like knowledge graphs. Additionally, there are plans to conduct in-depth research into the fusion of prior knowledge and the model, incorporating spatial location relationships among diseases or prior knowledge about disease occurrence time to achieve more accurate disease detection. Knowledge reasoning methods will be employed to express prior knowledge more effectively, and efforts will be made to further enhance the proposed method and apply it to a wider range of plant disease detection scenarios, aiming for more accurate multi-category plant disease detection.

## Data availability statement

The dataset and code in this study can be accessed by contacting the corresponding author.

## Author contributions

XW: Conceptualization, Investigation, Software, Writing – original draft, Writing – review & editing. JL: Funding acquisition, Methodology, Software, Validation, Visualization, Writing – original draft, Writing – review & editing.
